# An Ambispective Community-Based Survival Study of Common Cancers in Rural Jodhpur, Rajasthan, Western India

**DOI:** 10.7759/cureus.59990

**Published:** 2024-05-09

**Authors:** Garima Singh, Pankaja Raghav, Neeti Rustagi, Abhishek Jaiswal

**Affiliations:** 1 Department of Community Medicine, Lady Hardinge Medical College, Delhi, IND; 2 Department of Community Medicine and Family Medicine, All India Institute of Medical Sciences, Jodhpur, Jodhpur, IND; 3 Department of Community Medicine, Employee State Insurance Corporation Medical College and Hospital, Faridabad, IND

**Keywords:** breast neoplasms, head and neck neoplasms, cancers, mortality, survival

## Abstract

Background

Cancer is the leading cause of death globally. Information on cancer patterns and survival is essential for the effective planning and implementation of cancer control interventions.

Objective

This study aimed to identify various factors associated with the survival estimates of common cancers.

Methods

A community-based ambispective study was conducted in a rural population. Data were collected from individuals diagnosed with cancer or relatives of individuals who died of cancer. The total population covered was 82,983. All cancer cases diagnosed since 2005 and followed until the year 2020 were included. Survival analysis and five-year survival rates were estimated. A Cox proportional hazard model was used.

Results

A total of 146 cancer patients were included in the study. Five-year survival estimates for breast cancer, head and neck cancer, and GI cancer were 72%, 28%, and 0%, respectively. The median survival time was lowest for GI cancers (1 year), and for head and neck and breast cancers, it was 3 and 6 years, respectively. Multivariate Cox regression was performed, adjusting for age, type of hospital, alcohol use, tobacco use, opium use, gender, treatment sought, GI cancer, frequency of changing hospitals, and frequency of follow-up. After adjustment, changing hospitals ≥3 times, being lost to follow-up, receiving no treatment, tobacco abuse, and the presence of GI cancers were significantly associated with survival estimates.

Conclusions

The five-year survival estimate for GI cancers was the lowest compared to other cancers. Study participants who were lost to follow-up or who took no treatment were significantly associated with lower survival estimates.

## Introduction

Globally, cancer is the leading cause of death after cardiovascular diseases. In 2020, cancer resulted in approximately 10 million deaths [1]. The most common cancers that year were breast (2.26 million cases), lung (2.21 million cases), colon and rectum (1.93 million cases), prostate (1.41 million cases), non-melanoma skin cancer (1.20 million cases), and stomach (1.09 million cases). The leading causes of cancer death in 2020 were lung (1.80 million deaths), colon and rectum (935,000 deaths), liver (830,000 deaths), followed by stomach (769,000 deaths) and breast (685,000 deaths) [1]. Approximately 70% of cancer deaths occur in low- and middle-income countries [2], where late-stage presentation and limited access to diagnosis and treatment are common. Reports indicate that comprehensive therapy is available in more than 90% of high-income countries, but in fewer than 15% of low-income countries [3].

The estimated age-standardized mortality rate (world) according to the International Agency for Research on Cancer (IARC) for India in 2020 for all cancers, both sexes, all ages was 63.1 per 100,000 [4]. According to Indian population census data, mortality due to cancer in India was alarmingly high, with about 806,000 existing cases at the end of the last century [5], which increased to 851,678 deaths by the year 2020 [4]. Survival statistics are advantageous, principally as comparative estimates to show differences in survival over time between different populations and their subgroups. These estimates help us recognize possible explanations for the disparities and provide targets for improvement and a way to monitor progress towards these targets [6].

According to the IARC, the five-year age-standardized relative survival rates for carcinoma in developing countries were highest for the cervix (60%), followed by the breast (47%), oral cavity (36%), esophagus (9%), stomach (8%), and lung (6%) [7]. Among head and neck carcinomas, the five-year relative survival was highest for cancer of the lip (47%) and lowest for cancer of the hypopharynx (14%). Carcinoma of the stomach, pancreas, and esophagus had survival statistics of 10%, 9%, and 8%, respectively.

In males, the five-year relative survival was highest for cancer of the lip (52%) followed by Hodgkin lymphoma (38%), larynx, and oral cavity (37%). Survival from carcinoma of the lip was markedly higher among males compared to females (41%) [6]. The five-year relative survival for females was highest for carcinoma of the cervix (59%), followed by carcinoma of the breast (49%), Hodgkin lymphoma (47%), and carcinoma of the lip (41%). Survival statistics were higher among females (32%) compared to males (14%) for carcinoma of the tonsil [6].

Although the National Programme for Prevention and Control of Cancer, Diabetes, Cardiovascular Diseases, and Stroke (NPCDCS) was launched in 2010, there have been very few studies on survival estimates; those conducted were limited to urban areas of Rajasthan. Cancer data from Western India is also scarce. Most studies conducted in the past were based only on major hospitals and pathology centers [8]. Moreover, studies exploring cancer survival estimates are rare in India. With only one rural registry (Barshi rural registry, Maharashtra), data from the rural populations of other states is limited.

All India Institute of Medical Sciences (AIIMS) Jodhpur, established in 2012, provides cancer treatment to both rural and urban populations of Jodhpur. However, due to the lack of a population-based registry in Rajasthan, there was no proper information regarding the epidemiology and survival estimates of cancer, especially in rural areas. It was crucial to conduct such studies to understand cancer survival estimates in rural areas. Hence, the present community-based ambispective study was undertaken to determine the cancer survival rates in the rural population of Jodhpur. The current study aimed to analyze the survival estimates of common cancers over the past 15 years in the rural population of Jodhpur.

## Materials and methods

A community-based ambispective study was conducted in rural Jodhpur, Rajasthan. Jodhpur district is composed of 65.7% rural and 34.3% urban populations. The total rural population comprises 2,422,551 individuals (M: 1,260,328 and F: 1,162,223). Administratively, the rural areas of Jodhpur are divided into ten blocks. From the sampling frame of these ten blocks, the Mandore block was selected by simple random sampling. The rural area of Mandore block consists of 189,931 persons (M: 95,538 and F: 94,393) [9]. There are 113 villages in the selected block, out of which one-third of the total number of villages (37 villages) were included in the study.

The study included individuals with a diagnosed case of cancer or those who had died from cancer in the last 15 years in the selected villages. For cancer survivors suffering from more than one type of cancer, details regarding the cancer under treatment at the time of the visit were considered. In families with more than one member, all family members residing in the selected village were included in the study. In cases of death due to cancer, details were obtained from the caregivers.

The study participants were contacted after liaising with healthcare workers (Anganwadi workers (AWW), Accredited Social Health Activists (ASHA), Auxiliary Nurse Midwife (ANM)) and community leaders (both formal and informal) to identify cancer cases and deaths due to cancer in the study area. Caregivers with a diagnosed case of cancer or those who had experienced a death due to cancer in the last 15 years in selected villages were also contacted to collect information. Written informed consent was obtained from participants. Strict confidentiality was maintained. Apart from demographic details, information was collected about the type of cancer, year of diagnosis, year of death, any substance abuse, treatment sought, healthcare facilities from where treatment was received, and follow-up patterns. The information was collected from both cancer survivors and caregivers of deceased patients.

Operational definition

Cancer Case

A cancer case was defined as a participant who had a diagnosis of cancer (in situ or invasive) or a benign or borderline CNS tumor. This includes the use of terms commonly associated with cancer, such as "cancer," "malignant," "carcinoma," "sarcoma," "leukemia," and "lymphoma," by a recognized medical professional, or cases that have a confirmed positive histology, cytology, or positive microscopic of in situ or invasive cancer.

ICD Classification of Cancer

All neoplasms/tumors with the behavior code “3” (International Classification of Diseases - Oncology, third edition (ICD-O-3)) were included. This definition is used in cancer registries, pathology, and departments specialized in cancer. According to the International Classification of Diseases (ICD-10: C00-C97), neoplasms reported for all anatomical sites of cancer were included in the present study [10].

Ethics

Written informed consent was obtained from all participants willing to participate in the study. Data collected from the participants was de-identified to protect personal information. Ethical permission for the present study was granted by the Institute’s Ethics Committee, AIIMS, Jodhpur, Rajasthan, India, vide letter no. AIIMS/IEC/2018/576 dated 24/12/2018.

Statistical analysis

Data were entered into Microsoft Excel 2011, checked for errors, and cleaned before analysis. Categorical variables were reported as frequency and percentage. Continuous variables were reported as median and interquartile range (IQR). For the survival analysis, Kaplan-Meier estimates and life table methods were used. Survival curves for all variables were compared using the Log Rank test. All dependent variables with a p-value less than 0.25 in Log Rank tests were included in the Multivariable Cox Proportional Hazard Model. Hazard ratios were reported with 95% confidence intervals (CI). Statistical significance was established at a p-value < 0.05. Statistical analysis was performed using STATA version 16 (StataCorp. 2019. Statistical Software: Release 16. College Station, TX: StataCorp LLC). Identifiers were removed from the dataset before analysis.

## Results

The present study included 37 villages covering a population of 82,983 persons (M: 42,928 and F: 40,055), which included 146 cancer cases. Among the 146 study participants, 60.3% (88) were males. The ages of the study participants ranged from 6 to 84 years, with the majority (43.2%) being in the age group of 60-79 years. Most of the cancer patients were Hindu by religion (93.2%, 136). Among the 146 cancer patients, more than 40% (45.2%, 66) were alive. Participants' occupations were classified based on the Occupation Gazette of India 2020 [11]. Almost 44% of the participants were unemployed, which included homemakers (34.9%, 51) and students (3.4%, 5). Out of the total study subjects, 42.5% (62) were illiterate, while only 4.2% (6) were professionals. Socioeconomic status was calculated based on the modified BG Prasad Scale (2020) [12]. One-fourth (26.7%, 39) of the participants belonged to the upper-middle class, while 26.0% (38) belonged to the lower-middle class.

Univariate analysis of factors affecting cancer survival

Upon analysis of variables that affect the survival of cancer patients, the use of tobacco was significant (p-value = 0.042). Mortality was highest among participants with gastrointestinal cancers (p-value < 0.001). Graduate-level education was significantly associated (p-value = 0.018) with the survival estimates of cancer patients. The frequency of changing hospitals more than three times (p-value = 0.024) was significantly associated with the survival of cancer patients (Table 1).

**Table 1:** Hazard ratio of variables affecting cancer survival. *Head and Neck Cancer: Includes oral cancer, laryngeal cancer, and nasopharyngeal cancer. **Gastrointestinal Cancer: Includes tumors of the upper gastrointestinal tract (esophageal cancer, stomach cancer, small intestine cancer) and tumors of the lower gastrointestinal tract (colorectal cancer, tumors of the appendix). ***Others: Includes soft tissues and bone carcinoma, gynecological malignancies, primary brain tumors, tumors of the liver and biliary tree, bladder and renal cell carcinoma, lung cancer, skin cancer, testicular cancer, endocrine malignancies, and prostatic cancer.

Variable		Hazard Ratio (95% CI)	P-value
Age (in years, continuous)		1.02 (0.99-1.03)	0.065
Gender (Female)		0.91 (0.57-1.44)	0.678
Tobacco users (Yes)		1.59 (1.02-2.48)	0.042
Opium user (Yes)		1.24 (0.75-2.08)	0.399
Alcohol user (Yes)		1.20 (0.69-2.09)	0.505
Any substance abuse (Tobacco/Alcohol/Opium) (Present)		1.37 (0.86-2.18)	0.187
Comorbidity		0.84 (0.46-1.52)	0.56
Type of Cancer by anatomical location	Head and Neck Cancer*	Ref.	-
	Breast Cancer**	0.44 (0.18-1.05)	0.067
	GI Cancer	2.59 (1.44-4.65)	0.001
	Others***	0.78 (0.45-1.34)	0.373
Occupation	Professional	Ref.	-
	Semi Professional	0.76 (0.15-3.79)	0.744
	Clerical/Shop owner/Farm	1.62 (0.65-4.01)	0.3
	Skilled worker	1.28 (0.32-5.14)	0.724
	Semiskilled worker	1.41 (0.17-11.7)	0.751
	Unskilled worker	2.55 (0.94-6.9)	0.067
	Unemployed	1.58 (0.66-3.75)	0.306
Education	Professional	Ref.	-
	Graduate	0.13 (0.02-0.71)	0.018
	Intermediate/Diploma	0.39 (0.12-1.27)	0.119
	High School	0.45 (0.12-1.68)	0.233
	Middle School	0.57 (0.17-1.8)	0.355
	Primary School	0.56 (0.16-1.93)	0.359
	Illiterate	0.78 (0.27-2.19)	0.635
Socio economic status	I (Upper class)	Ref.	-
	II (Upper middle class	1.41(0.64-3.10)	0.387
	III (Middle)	1.36 (0.59-3.11)	0.47
	IV (Lower middle class)	1.64 (0.76-3.55)	0.208
	V (Lower class)	1.07 (0.44-2.59)	0.877
Health care facility	Government	Ref.	-
	Private	1.26 (0.69-2.31)	0.443
Place for seeking treatment currently	Jodhpur	Ref.	-
	Outside Jodhpur	0.93 (0.56-1.54)	0.769
Frequent changes in hospitals	No change of hospital	Ref.	-
	More than once	0.96 (0.60-1.55)	0.874
	More than twice	0.58 (0.21-1.65)	0.314
	More than thrice	1.06 (0.25-4.4)	0.934
Marital status	Married	Ref.	-
	Never married	0.54 (0.132-2.20)	0.391
Preference for treatment	Only allopathic	Ref.	-
	Alternate medicine + Allopathy	1.54 (0.97-2.47)	0.068
	No treatment taken	3.78 (1.58-9.07)	0.003
Family type	Nuclear	0.77 (0.49-1.22)	0.274
	Joint	Ref.	-
Follow-up after treatment	Regular follow-up	Ref.	-
	Irregular follow-up	1.85 (0.99-3.46)	0.053
	Lost to follow-up	1.81 (1.08-3.04)	0.024

Survival analysis and Kaplan-Meier estimates

For the survival analysis, a life table was prepared, as shown below in Table 2. Total time in years is denoted in column (I), starting from 6 months to 13 years. The number of cancer patients entered into the study each year is noted in column (II). Patients who survived are recorded in the next column (III), and the number of terminal events is mentioned in column (IV). The number of deceased patients for each year, divided by patients at risk, was calculated as the survival proportion, shown in column (V). Based on the survival proportion, the cumulative survival proportion for each year was calculated. The median survival time was calculated as 3 years.

**Table 2:** Life table analysis.

I	II	III	IV	V	VI	VII
Time (year)	Total patients	Patient surviving	No. of Terminal Events	Proportion Surviving	Cumulative Proportion Surviving	95% CI
0.5	146	2	21	0.86	0.86	0.78-0.90
1	123	17	26	0.77	0.66	0.57-0.73
1.5	80	0	0	1	0.66	0.57-0.73
2	80	22	15	0.78	0.52	0.42-0.60
2.5	43	0	0	1	0.52	0.42-0.60
3	43	10	11	0.71	0.37	0.27-0.46
3.5	22	0	0	1	0.37	0.27-0.46
4	22	5	2	0.9	0.33	0.23-0.43
4.5	15	0	0	1	0.33	0.23-0.43
5	15	5	0	1	0.33	0.23-0.43
5.5	10	0	0	1	0.33	0.23-0.43
6	10	4	2	0.75	0.25	0.13-0.37
6.5	4	0	0	1	0.25	0.13-0.37
7	4	1	0	1	0.25	0.13-0.37
7.5	3	0	0	1	0.25	0.13-0.37
8	3	1	0	1	0.25	0.13-0.37
8.5	2	0	0	1	0.25	0.13-0.37
9	2	0	0	1	0.25	0.13-0.37
9.5	2	0	0	1	0.25	0.13-0.37
10	2	0	1	0.5	0.12	0.01-0.35
10.5	1	0	0	1	0.12	0.01-0.35
11	1	0	0	1	0.12	0.01-0.35
11.5	1	0	0	1	0.12	0.01-0.35
12	1	0	0	1	0.12	0.01-0.35
12.5	1	0	0	1	0.12	0.01-0.35
13	1	0	1	0	0	

**Figure 1:**
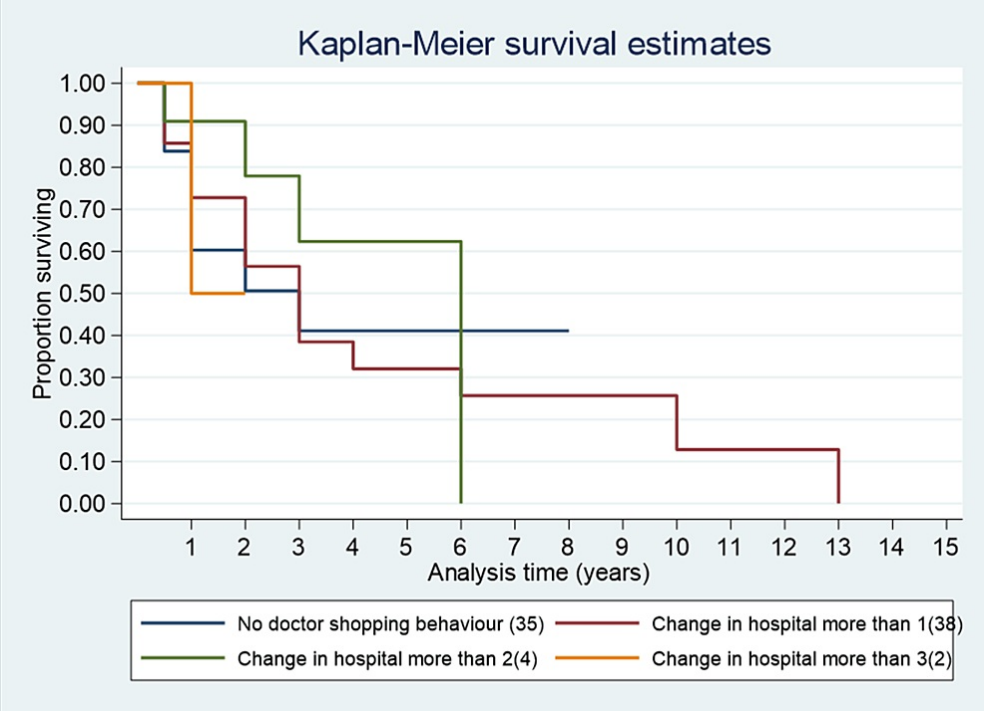
Survival curve for patients with behaviour of frequent change of hospitals.

**Figure 2:**
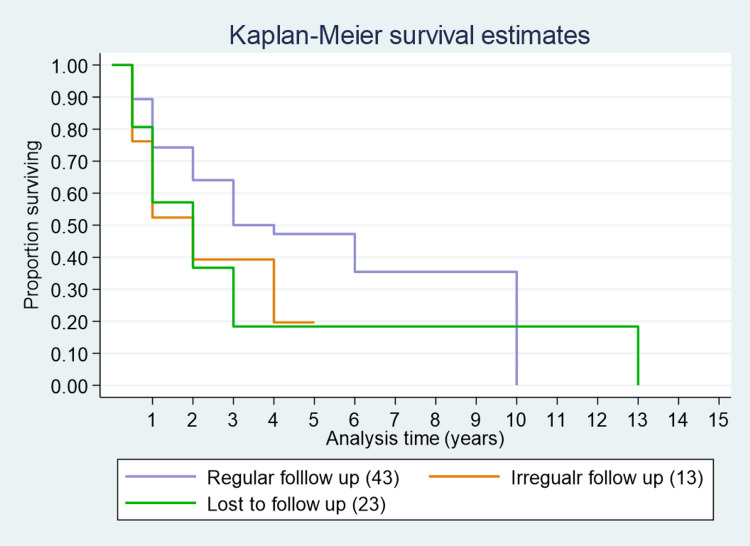
Survival curve for follow-up among cancer patients.

**Figure 3:**
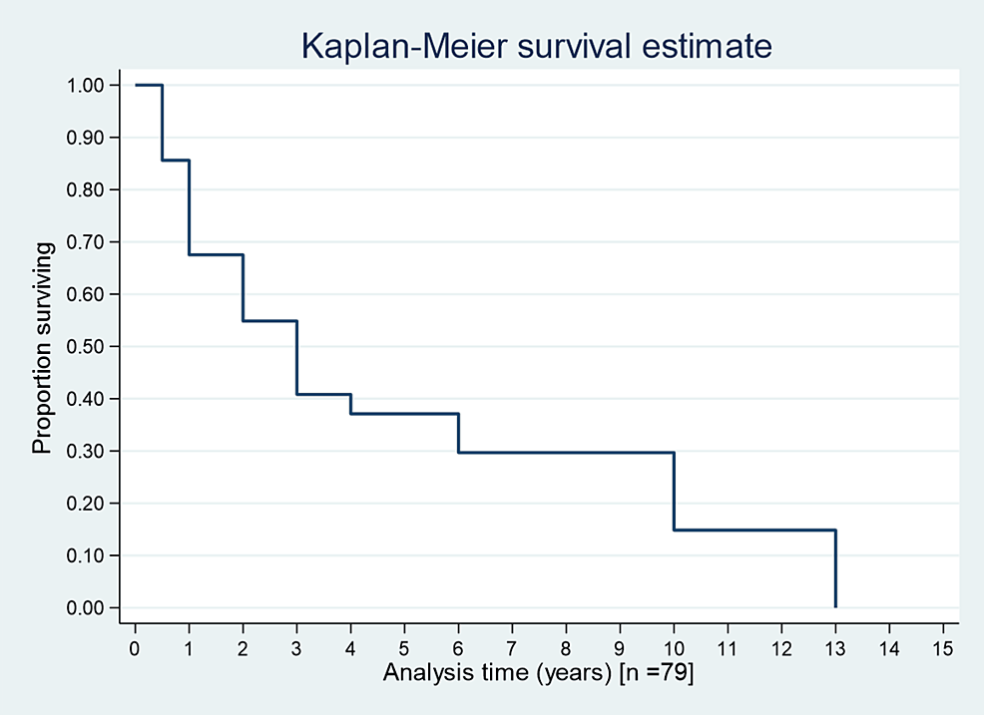
Overall survival estimates for 15 years.

**Figure 4:**
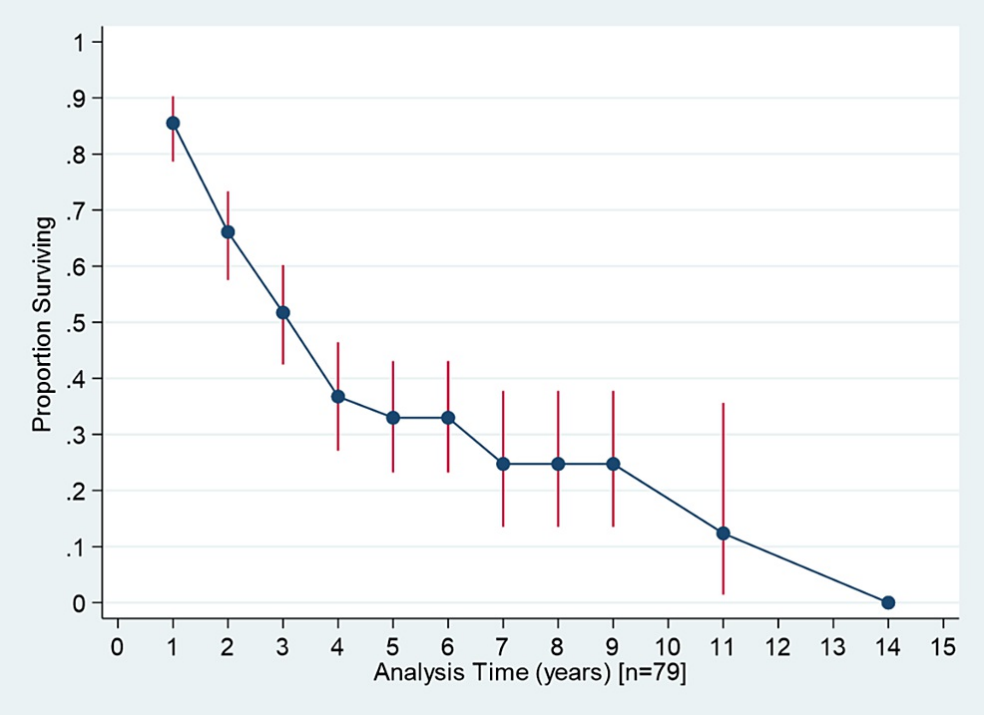
Year-wise survival estimates for all the cancers.

The median survival time among cases who changed their hospital more than three times was one year; for those changing more than two times, it was six years; and for those changing more than once, it was three years (Figure 1). The median survival time for cancer cases adopting regular follow-up post-treatment was three years, whereas it was two years for both irregular follow-up and those lost to follow-up (Figure 2). Figure 3 shows the overall survival for 146 cancer cases over 15 years. Figure 4 presents one-year, three-year, and five-year survival estimates at 86% (95% CI 78.6%-90.31%), 37% (95% CI 27.09%-46.44%), and 33% (95% CI 23.20%-43.09%), respectively. 

**Figure 5:**
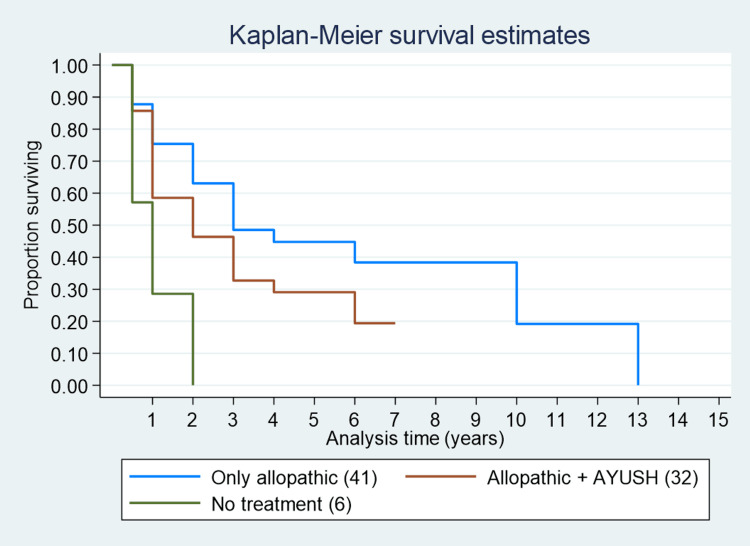
Survival curve for different modes of treatment.

The median survival of cases who did not receive cancer therapy after diagnosis was one year. For those whose preferred mode of treatment was Ayurveda, Yoga & Naturopathy, Unani, Siddha, and Homeopathy (AYUSH) over allopathic, it was two years. Cases with preferred allopathic treatments (radiotherapy/chemotherapy/surgery) had a median survival time of four years (Figure 5). The median survival time for those using complementary alternative medicine was two years, three years for those using only allopathic treatment, and one year for those not taking any treatment. The one-year, three-year, and five-year survival for those who were taking no treatment was 40%, 30%, and 0%, respectively. Survival estimates for those not taking treatment were significantly low (p=0.0017). 

For those using complementary alternative medicine, one-year, three-year, and five-year survival was 87%, 38%, and 35%, respectively, and for those using only allopathic medicine, it was 88%, 48%, and 45%, respectively. Participants who did not take any treatment (HR=3.78, 95% CI (1.58-9.07)) and those who took alternative medicine along with allopathic medicine (HR=1.54, 95% CI (0.97-2.47)) had a higher risk of death compared to those who took allopathic medicine only.

**Figure 6:**
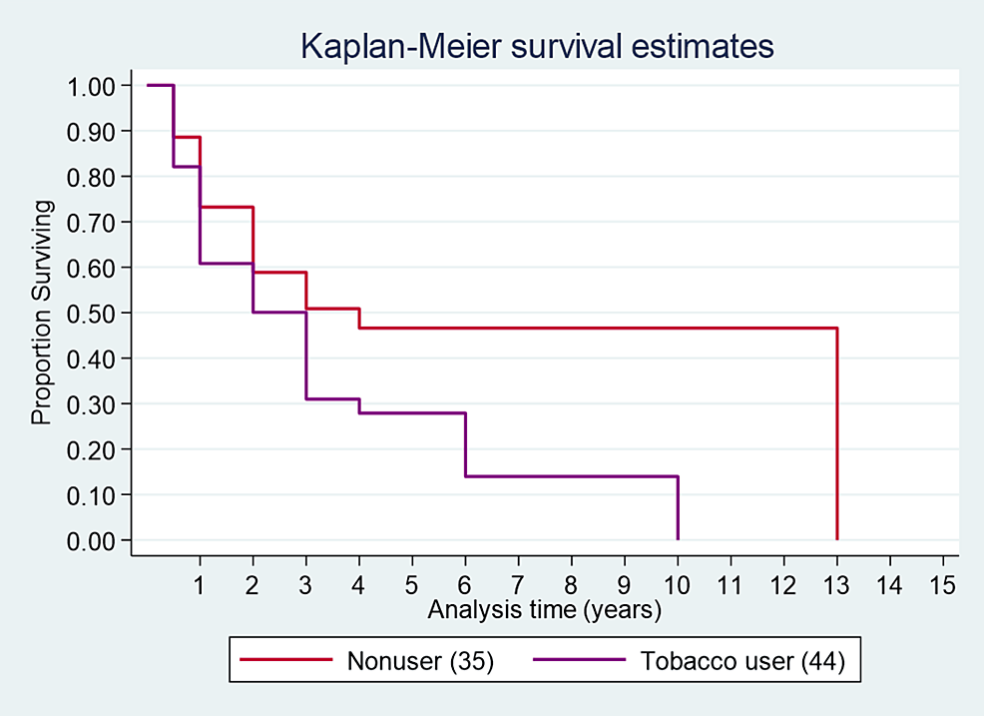
Survival curve for tobacco users.

The median survival time for individuals who consumed tobacco (two years) was 50% less than for those who did not consume tobacco (four years) (Figure 6). One-year, three-year, and five-year survival estimates for those who consumed tobacco were 60%, 30%, and 28% respectively, while for those who did not consume tobacco, the estimates were 73%, 50%, and 47%, respectively.

**Figure 7:**
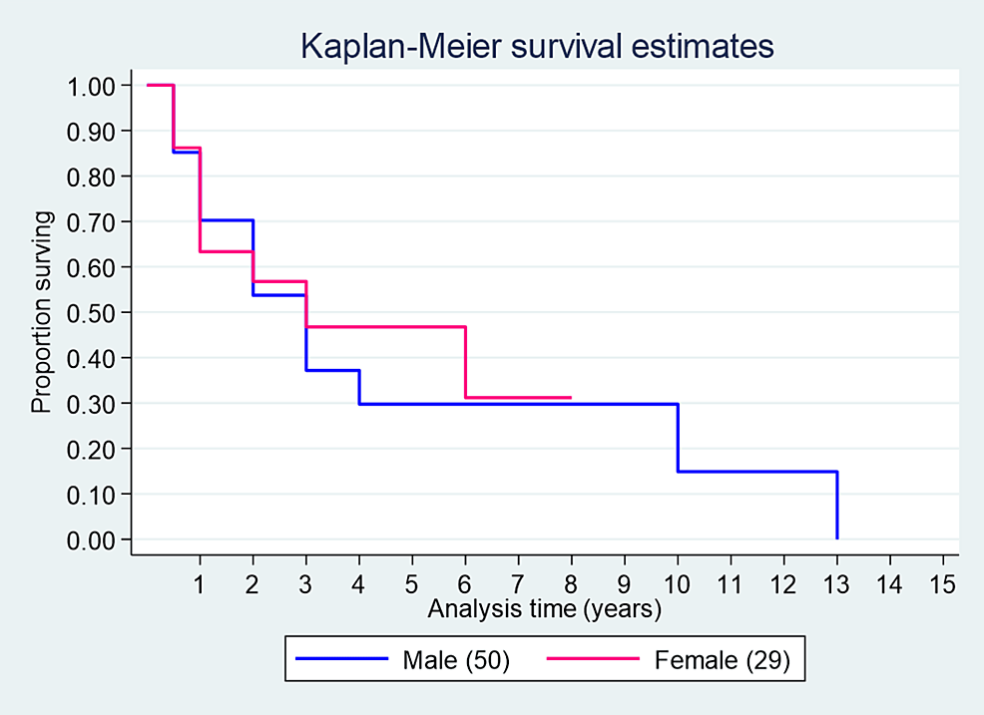
Survival curve among males and females.

One-year, three-year, and five-year survival estimates for females were 62%, 48%, and 48%, whereas for males, they were 70%, 38%, and 30%, respectively (Figure 7).

**Figure 8:**
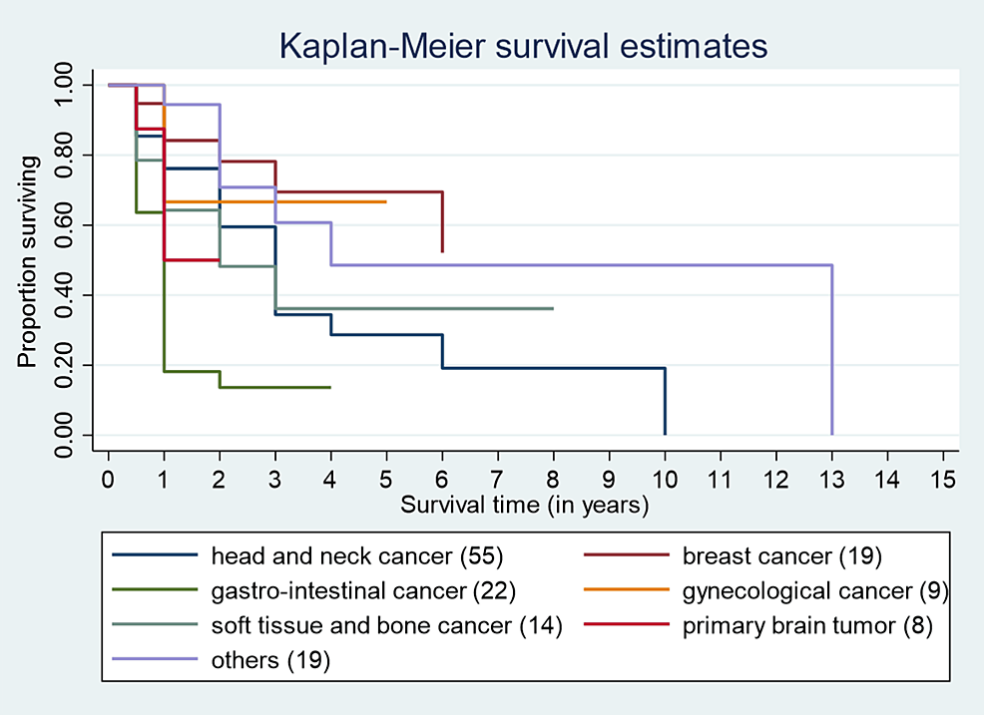
Survival curve for different types of cancers.

The median survival time for GI cancers was the lowest at one year, while for head and neck cancer, it was three years, and for breast cancer, it was six years (Figure 8). The median survival times for primary brain tumors and bone cancers were one year and three years, respectively. One-year, three-year, and five-year survival estimates for head and neck cancer were 87%, 65%, and 28% respectively. For breast cancer, they were 87%, 72%, and 72% respectively. Meanwhile, for gastrointestinal cancer, the estimates were 20%, 17%, and 0% respectively, with none of the patients surviving for five years. The one-year survival for primary brain tumors was 50%, for gynecological malignancies it was 70%, while for soft tissue sarcomas and bone cancers it was 65%. 

Appendix 1 shows the patients entering the study at different time points.

Patients suffering from gastrointestinal cancers had the highest mortality (p-value < 0.0001). Survival in cancer patients who were not taking treatment was significantly lower (p-value = 0.0017). Substance abuse among cancer patients was also significantly associated (p-value = 0.0329) with lower survival rates (Table 3).

**Table 3:** Comparison of survival curves (log rank test).

Variables		Observed death (n)	Expected death (n)	Chi-Square	P-value
Age (in years)	≤ 40	8	16.03	6.23	0.0126
	> 40	71	62.97		
Gender	Male	50	48.22	0.21	0.6468
	Female	29	30.78		
Alcohol use	No	63	65.24	0.55	0.4598
	Yes	16	13.76		
Tobacco use	No	35	43.99	5.10	0.0239
	Yes	44	35.01		
Opium use	No	59	62.06	0.87	0.3503
	Yes	20	16.94		
Type of Hospital	Govt.	68	65.69	0.59	0.4413
	Private	11	13.31		
Treatment sought	No	11	4.93	9.80	0.0017
	Yes	68	74.07		
GI cancer	Other than GI Cancer	60	71.52	24.4	<0.0001
	Present	19	7.48		

Multivariable analysis

Multivariable Cox regression was carried out, adjusting for age, type of hospital, alcohol use, tobacco use, opium use, gender, treatment sought, gastrointestinal cancer, frequency of hospital changes, and frequency of follow-up. After adjusting, the frequency of hospital changes, follow-up frequency after treatment, absence of treatment, tobacco use, and gastrointestinal cancer were found to be significantly associated with survival (Table 4).

**Table 4:** Cox regression: the proportional hazard model.

Variables		Hazard Ratio (Unadjusted)	95% CI	Hazard Ratio (Adjusted)	95% CI
Age (in years)	(Continuous)	1.02	0.99-1.03	1.01	0.99-1.03
Type of Hospital	Govt.	Ref.	-	Ref.	-
	Private	0.80	0.42-1.51	0.78	0.41-1.49
Alcohol use	No	Ref.	-	Ref.	-
	Yes	1.21	0.69-2.10	1.29	0.68-2.47
Tobacco use	No	Ref.	-	Ref.	-
	Yes	1.59	1.02-2.49	1.81	1.03-3.14
Opium use	No	Ref.	-	Ref.	-
	Yes	1.25	0.75-2.08	1.21	0.62-2.35
Gender	Male	Ref.	-	Ref.	-
	Female	0.91	0.57-1.44	1.82	0.89-3.67
Treatment sought	No	Ref.	-	Ref.	-
	Yes	0.41	0.21-0.77	0.41	0.21-0.81
GI cancer	No	Ref.	-	Ref.	-
	Yes	3.18	1.87-5.41	3.31	1.90-5.76
Frequency of change in hospital	More than once	0.96	0.60-1.55	1.03	0.57-1.85
	More than twice	0.58	0.21-1.65	0.74	0.24-2.29
	More than thrice	1.06	0.25-4.4	2.96	0.65-13.6
Frequency of follow-up	Regular follow-up	Ref	-	Ref	-
	Irregular follow-up	1.85	0.99-3.46	2.06	1.03-4.11
	Lost to follow-up	1.81	1.08-3.04	2.10	1.10-3.99

## Discussion

In this study, the one-year, three-year, and five-year survival estimates for head and neck cancer were 87%, 65%, and 28%, respectively, whereas the median survival estimate was three years. Saxena PP et al. [13] conducted survival analysis on head and neck cancer in a hospital-based registry in Karnataka, India, and reported that five-year survival ranged from 25% for tongue cancer to 74% for lip cancer.

For breast cancer, the one-year, three-year, and five-year survival rates in the present study were similar to those reported in different registries in India (45-55%) [14-17]. Abedi G et al. conducted a study that reported one-year, three-year, five-year, and ten-year survival rates for breast cancer in Iran, with findings slightly higher than those typically observed. The probable reasons for the slight increase in survival rates could be the availability of awareness programs, public training, and facilities for diagnosis and treatment.

None of the patients survived for five years for gastrointestinal cancers in the current research. Pisani B et al. [18] also reported low survival rates for gastrointestinal cancers. The one-year survival rate for gynecological malignancies in the current study was 70%, which was comparable with the survival estimates of the Barshi rural registry [19].

Tobacco-related cancers (TRC) include lip, tongue, mouth, oropharynx, hypopharynx, larynx, lung, esophagus, stomach, pancreas, and urinary bladder cancers. The study conducted by Vendhan G et al. [20] included all TRCs and reported that the five-year relative survival rates for tobacco-related cancer sites were as follows: lip (46%), tongue (26%), mouth (33%), oropharynx (21%), hypopharynx (18%), larynx (39%), esophagus (7%), stomach (8%), pancreas (5%), lung (8%), and urinary bladder (23%) [21]. Forty-eight percent and nineteen percent of all cancers among males and females in India, respectively, are tobacco-related [20].

The study conducted by Johnson SB and Yu BJ [21] on the national cancer database in the United States reported that patients who refused the recommended cancer treatments in favor of alternative medicine (AM) had a higher risk of mortality (HR: 2.50, 95% CI: 1.88-3.27). In the same study, out of 1,901,815 patients, 258 used complementary medicine. The usage of complementary medicine was associated with a lower five-year overall survival estimate compared to those patients who did not use complementary medicine (82.2% (95% CI: 76.0%-87.0%) vs. 86.6% (95% CI: 84.0%-88.9%); p-value = 0.001) and had a higher risk of mortality (HR 2.08, 95% CI 1.50-2.90) [22]. Risberg T et al. conducted a follow-up study on 515 cancer patients in Norway in 2003 and reported that death rates were higher among AM users (79%) than among those who did not use AM (65%). The hazard ratio of death for any use of AM compared with no use of AM was 1.30, (95% CI: 0.99 - 1.70; P=0.056), suggesting that AM use may predict shorter survival [22].

The five-year survival estimates for patients who were lost to follow-up, post-treatment in the current study, was 23%. These results were concordant with the study done by Swaminathan et al., which reported a 5-year survival estimate of 22% for patients who were lost to follow-up [16].

Limitations

As this is a cross-sectional study, only risk factors were identified, and no temporal associations could be determined. The median survival time calculated in the study was overall for all types of cancers combined, and for groups of cancers. Cancer-specific survival was not estimated. Since the patients or primary caregivers had to recollect information over a long period of time for the questions posed, the chances of recall bias in the present study cannot be overlooked.

## Conclusions

In conclusion, the survival and prognosis of a particular cancer are affected by multiple factors. Such as type of cancer, cancer stage, treatment sought, use of tobacco, and loss to follow-up to health care facilities after initiation of treatment were significantly associated with low survival estimates. Policymakers should take these findings into account and use them to inform future activities. Further research into these elements may be able to shed light on the subject and increase the effectiveness of future cancer policies. As most of the participants preferred public health systems, government healthcare facilities should be strengthened for primary-level care and initial treatment at the rural level.

There is a need to establish a referral mechanism. Community-based screening should be conducted to generate awareness about the screening program and its role in cancer prevention. Additionally, training and monitoring sessions for ASHAs, ANMs, and Anganwadi workers should be conducted to strengthen ongoing activities.

## Figures and Tables

**Figure 9:**
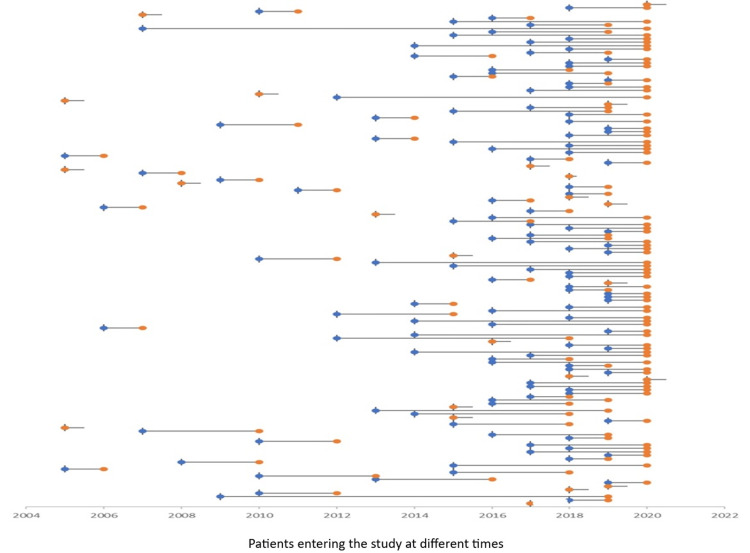
Appendix showing patients entering the study at different times with known and censored survival times. A blue dot indicates the time of entry into the study, and an orange dot indicates either death due to cancer or the end of the study.

## Appendices

Appendix 1 
